# Molecular prevalence and genotype identification of *Enterocytozoon bieneusi* in cattle and goats from Zhejiang Province, China

**DOI:** 10.3389/fvets.2024.1415813

**Published:** 2024-11-19

**Authors:** Xianming Xin, Lijie Sun, Wei Liu, Jiayin Zhang, Shiyang Ma, Xinyi Fu, Wei Zhao, Baolong Yan

**Affiliations:** ^1^School of Basic Medical Sciences, Wenzhou Medical University, Wenzhou, China; ^2^Department of Clinical Laboratory, The Fifth Affiliated Hospital, Sun Yat-sen University, Zhuhai, China; ^3^School of Public Health and Management, Wenzhou Medical University, Wenzhou, China

**Keywords:** *E. bieneusi*, cattle, goat, genotype, zoonotic, China

## Abstract

**Introduction:**

*Enterocytozoon bieneusi* (*E. bieneusi*) is a widespread intracellular fungi that poses a significant zoonotic threat due to its infectivity toward both humans and animals.

**Methods:**

To evaluate the zoonotic transmission potential of this fungi, a molecular investigation was undertaken on *E. bieneusi* in cattle and goats reared across multiple cities in Zhejiang Province, China. A total of 651 fresh samples were collected, consisting of 265 cattle and 386 goats. The presence of *E. bieneusi* was determined by PCR amplification and sequencing analysis of the internal transcribed spacer (ITS) region of the small subunit ribosomal RNA (*SSU rRNA*) gene in all collected samples.

**Results:**

The results revealed that 17.1% (111/859) of the animals were afflicted with *E. bieneusi*, cattle having a prevalence of 14.0% (37/265) and goats displaying a higher rate of 19.2% (74/386). Seventeen *E. bieneusi* genotypes were identified, including 10 known, CHG5 (*n* = 30), CHG3 (*n* = 21), I (*n* = 14), J (*n* = 12), CHG2 (*n* = 11), COS-II (*n* = 8), D (*n* = 4), CHG19 (*n* = 2), ETMK5 (*n* = 1), and Henan III (*n* = 1), and seven novel, ZJG-I to ZJG-VI and ZJN-I (one each) genotypes.

**Discussion:**

These findings indicate widespread infection of *E. bieneusi* among the surveyed animals, thereby raising concerns about zoonotic genotypes that could pose potential threats to public health. Furthermore, the identification of novel genotypes of *E. bieneusi* offers valuable insights into the genetic diversity of this pathogen.

## Introduction

Microsporidia are eukaryotic fungi residing within the cells of their hosts. They encompass a diverse range of over 1,500 species, with at least 17 species confirmed to infect humans (1, 2). *Enterocytozoon bieneusi* is the leading cause of microsporidiosis among humans, constituting over 90% of all microsporidiosis cases globally (3). In immunocompetent individuals, infections typically resolve within weeks or months, but asymptomatic infections can persist. However, individuals with compromised immune systems are at risk of developing fatal microsporidiosis caused by *E. bieneusi* (4). Additionally, *E. bieneusi* has also been found in diverse animals, including domestic and wild species, with evidence of cross-transmission between humans and animals (5). The reported outbreaks of food-borne and waterborne diseases have underscored the gravity of the threat posed by *E. bieneusi* (6, 7). Therefore, the National Institute of Allergy and Infectious Diseases (NIAID) has designated it as a Category B priority pathogen, while the Environmental Protection Agency (EPA) of the United States has identified it as a potential waterborne contaminant (8).

*Enterocytozoon bieneusi*'s genotyping tools and phylogenetic analysis are vital for gaining insights into its host specificity and transmission routes (9). The internal transcribed spacer (ITS) region of the small subunit ribosomal RNA (*SSU rRNA*) gene in *E. bieneusi* exhibits sequence variations, rendering it a dependable marker for molecular epidemiological research and a vital instrument for genetic analysis (10). Over 860 unique genotypes of *E. bieneusi* have been documented across 210 species of animals in 50 countries. Of these, 126 genotypes are exclusively human-associated, 614 are exclusively animal-associated, and a remarkable 58 genotypes are shared between humans and animals, indicating potential zoonotic transmission (8). All the genotypes were organized into 13 distinct groups, with the majority of human-infecting or zoonotic genotypes belonging to the first and second groups, thus highlighting their significance as potential zoonotic clusters (11–13). The remaining 11 clusters primarily reside in specific hosts and wastewater (11).

The genotypes within Group 1, such as D, EbpC, Type IV, and EbpA, as well as those within Group 2, specifically BEB6, I, and J, emerge as the most common zoonotic genotypes (8). The presence of these genotypes among pigs, cattle, and goats is frequently observed (8). For instance, 120 *E. bieneusi* genotypes have been identified in pigs, with EbpC and EbpA being the most common (14). Similarly, 41 genotypes have been documented in goats, primarily BEB6, while 97 genotypes have been reported in cattle, with genotypes I and J being the most frequent (15, 16). Therefore, pigs, cattle, and goats are crucial reservoirs of *E. bieneusi*, and infection with this microorganism is not just a veterinary concern; it also poses a significant public health risk. Regular monitoring of *E. bieneusi* in these animals is imperative to mitigate this risk.

China has firmly established its position as a leading authority in molecular epidemiological data related to *E. bieneusi* in cattle and goats (14–16). These studies, conducted across at least 21 provinces, have provided valuable insights into the transmission routes and infection sources of *E. bieneusi* (Tables 1, 2). However, significant data gaps persist in certain regions of China. For instance, in Zhejiang Province, no data are available regarding the infection of some commonly farmed animals, such as cattle and goats, with *E. bieneusi*. Therefore, the present study aimed to conduct a molecular investigation of *E. bieneusi* in goats and cattle in Zhejiang Province, China. The objectives of this study were to determine the infection rates and genotype composition and, ultimately, to assess the risk of zoonotic transmission of *E. bieneusi* carried by these animals at the genotype level.

**Table 1:** Infection rate and genotype divisions of *E. bieneusi* in cattle from China.

**Province**	**Positive/examined (%)**	**Genotype (*n*)**	**References**
Anhui	40/955 (4.2)	J (26), AHDC1 (3), AHYC2 (2), AHYC3 (2), AHYC6 (2), AHYC1 (1), AHYC4 (1), AHYC5 (1), AHYC7 (1), CHN11 (1)	(63)
Gansu	320/1,414 (22.6)	J (155), I (126), CGC2 (8), CGC1 (6), BEB4 (5), CM19 (5), BEB10 (3), CM21 (1), CGC3 (11)	(65)
Guangdong	221/1,828 (12.1)	J (117), I (91), D (7), BEB4 (3), EbpC (2), J/D (1)	(39, 65)
Hainan	31/314 (9.9)	EbpC (14), BEB4 (12), J (2), I (1), CHG5 (1), HNC-I (1)	(31)
Heilongjiang	217/10,046 (2.2)	J (81), I (44), BEB4 (33), O (26), EbpA (2), CS-4 (3), CHN-DC1 (2), CHN-DC2 (2), CHN-DC3 (2), D (1), EbpC (1), G (1), BEB4/J (1), CS-4/EbpC (1), CS-4/NECA1 (1), CS-4/NECA3 (1), EbpC/NECA2 (1), I/J (3), NECA4/NECA5 (1)	(18, 40–43)
Hebei and Tianjin	202/1,040 (19.4)	I (87), J (83), BEB4 (18), CHC8 (7), BEB6 (3), N (1), Ebpc (1), CHC6 (1), CHC7 (1)	(44)
Henan and Ningxia	375/1,497 (25.1)	J (172), I (81), BEB4 (53), CM8 (18), BEB6 (17), EbpC (6), COS-1 (5), D (2), EbpA (2), H (1), O (1), BEB8 (1), CD6 (1), CHG2 (1), CHG3 (1), CHC1 (1), CHC2 (1), CHC3 (1), CHC4 (1), CHC5 (1), NX1 (1), NX2 (1), NX3 (1), NX4 (1), NX5 (1), NX6 (1), NX7 (1), NX8 (1)	(45–47)
Inner Mongolia	28/108 (25.9)	J (14), I (9), BEB4 (3), COS-I (2)	(48)
Jiangxi	30/556 (5.4)	D (10), I (5), J (4), IV (4), N (1), BEB4 (1), JX-I (1), JX-II (1), JX-III (1), JX-IV (1), JX-V (1)	(49)
Jiangsu	177/1,366 (13.0)	J (138), I (21), BEB4 (10), J/I (5), J/BEB4 (1), TypeIV (1), CHC17 (1)	(65)
Jilin	35/93 (37.6)	CHN3 (12), CHN1 (6), I/J (5), I (2), J (2), CHN1/CHN3 (1), I/J/CHN1 (1), CHN1/CHN4 (1), J/CHN1 (1), CHN3/CHN4 (1)	(36)
Qinghai/Yunnan	15/898 (17.5)	J (6), COS I (3), BEB6 (1), PN (1), I (1), BEB4 (1), YNDCEB-90 (1), YNDCEB-174 (1)	(50, 51)
Shanxi	90/401 (22.4)	I (50), BEB4 (20), J (10), BEB8 (4), BEB6 (3), PigSpEb2 (1), CSC1 (1), CSC2 (1)	(63)
Shannxi	73/371 (19.7)	I (40), J (30), CHN1 (1), CSX1 (1), CSX2 (1)	(52)
Shanghai	214/809 (26.5)	J (145), BEB4 (59), CHN4 (4), Type IV and BEB4 (4), CHN15 (1), Mixed infection (1)	(53)
Shandong	24/148 (2.9)	J (20), I (2), BEB4 (2)	(45, 65)
Tibet	11/442 (2.5)	I (7), EbpC (2), CHC8 (2)	(54)
Xinjiang	215/764 (28.1)	J (165), I (19), EbpC (11), PigEBITS5 (5), BEB4 (4), CHV4 (3), D (2), CHC3 (1), CS-9 (1), KIN-1(1), CH5 (1), CAM5 (1), CC4 (1)	(70)

**Table 2:** Infection rate and genotype divisions of *E. bieneusi* in goats from China.

**Province**	**Positive/examined (%)**	**Genotype (*n*)**	**References**
Anhui	36/787 (4.6)	CHG3 (15), CHG1 (12), AHG2 (2), AHG1 (1), COS-II (1), BEB6 (1), CHG5 (1)	(55, 69)
Chongqing	5/8 (62.5)	CHG1 (2), CHG3 (1), CD6 (1), CHG12 (1)	(55)
Gansu	1/10 (10.0)	CHG3 (1)	(56)
Guangdong	40/226 (17.7)	CHG3 (32), CM21 (4), CHG1 (2), ET-L2 (2)	(57)
Guizhou	12/45 (26.7)	BEB6 (5), CHG1 (4), CHG3 (3)	(56)
Hainan	110/428 (25.7)	CHG5 (54), CHG3 (39), CHG2 (4), CM21 (3), D (2), AHG1 (1), HNG-I (1), HNG-II (1), BEB6 (4), CHG28 (1)	(56, 69)
Henan	290/643 (45.1)	BEB6 (73), CHG3 (70), CD6 (30), COS-I (15), CHG1 (15), CHS7 (14), CHG2 (10), CHG5 (6), E (3), F (2), KIN-1 (2), D (2), J (1), CHG6 (1), CHG7 (1), CHG8 (1), CHG9 (1), CHG10 (1), CHG11 (1), CHG13 (1), CHG18 (1), CHG20 (1), CHG21 (1), CHG22 (1), CHG23 (1), CHG25 (1), CHG26 (1), CHG27 (1), CHG28 (1), CHC8 (1), peru6 (1)	(55, 56, 58)
Heilongjiang	12/55 (21.8)	Peru6 (3), BEB6 (3), D (2), EbpC (2), EbpA (1), COG-I (1)	(59)
Jiangsu	25/125 (20.0)	CHG3 (12), BEB6 (7), CHG1 (2), CHG2 (2), CHG5 (1), CHG28 (1)	(56, 66)
Inner Mongolia	46/561 (8.2)	COS-I (17), BEB6 (15), CHG1 (8), BEB4 (2), NCF2 (1), CNR1 (2), CNR2 (1)	(48)
Ningxia^a^	237/660 (36%)	BEB6 (91), CHG3 (47), CM7 (38), CHG1 (18), CD6 (3), CHS8 (2), CHG5 (1), SX1 (1)	(60)
Shaanxi	240/733 (32.7)	CHG3 (61), CHG1 (73), BEB6 (18), CHG5 (9), CHG2 (6), SX1 (60), CHG14 (1), CHG16 (1), CHG24 (1), CHG28 (1), COS-II (1), CD6 (4), E (1), F (1)	(55, 58, 64, 68)
Tibet	25/260 (9.6)	EbpA (15), EbpC (16)	(61)
Yunan	123/1,041 (11.8)	CHG1 (28), Wildboar3 (16), BEB6 (29), CHG3 (13), CHG5 (8), D (4), J (2), CD6 (3), CHG28 (2), CYG-2 (2), CYG-3 (2), CHG2 (1), SDD1 (1), CYG-1 (1), CYG-4 (1), CHG16 (1), CHG17 (1), CHG19 (1), E (4), F (1), COS-I (1)	(55, 62)

^a^The data from Ningxia encompasses both sheep (41.1%, 148/360) and goats (29.7%, 89/300). However, the precise genotype details of goats and sheep remain indistinguishable.

## Materials and methods

### Ethics statement

The present study protocol involved rigorous scrutiny and secured approval from the Research Ethics Committee at Wenzhou Medical University, with reference number SCILLSC-2021-01. Additionally, the collection of all fecal samples was carried out with explicit consent from the owners or managers of the animals, and no harm was caused to the animals during the sample collection process.

### Collection of fecal specimens

**Figure 1:**
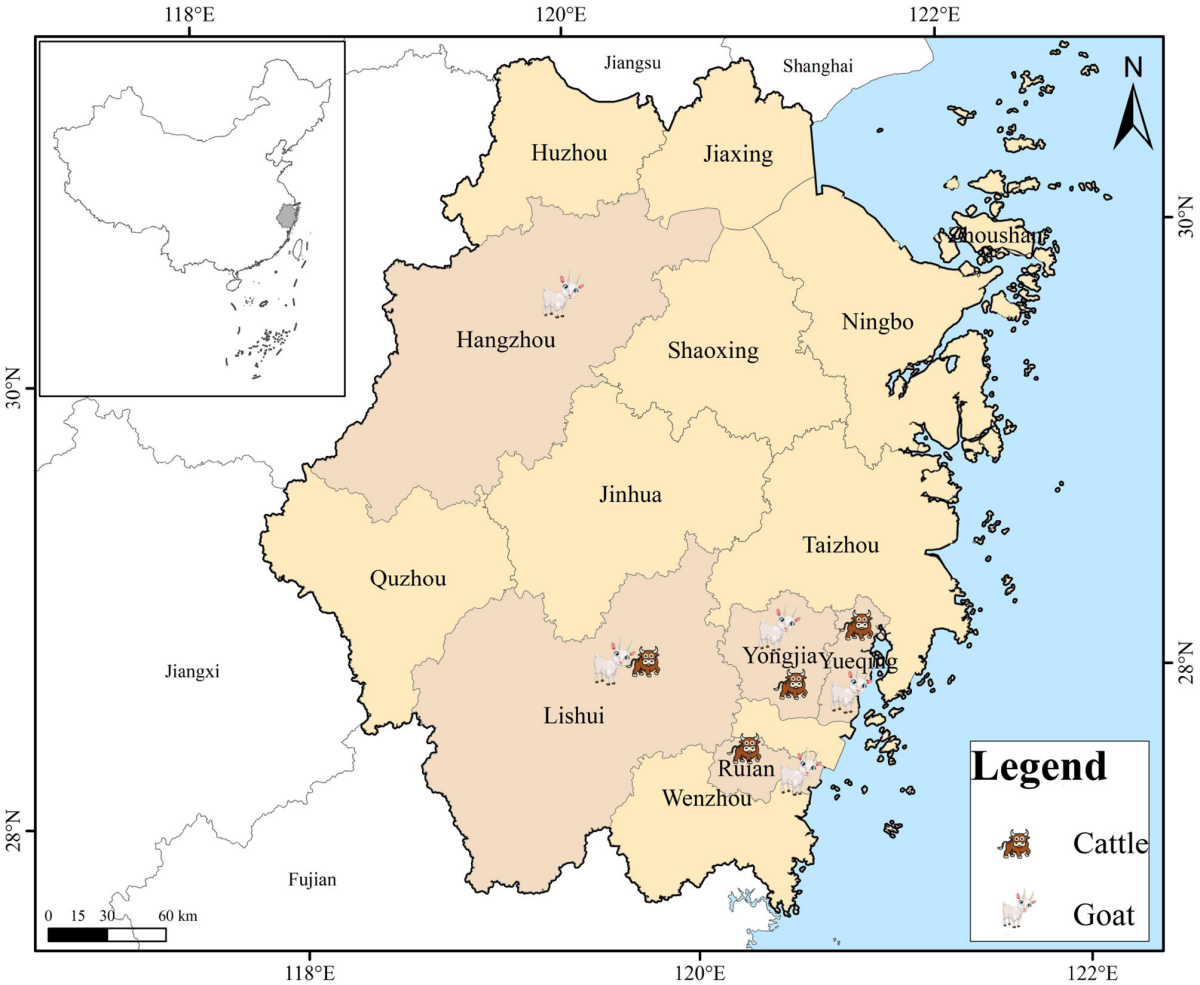
A map depicting the sampling locations of cattle and goats in Zhejiang Province, China.

Between September 2021 and May 2023, an extensive collection of 651 fresh fecal samples was conducted, encompassing 265 cattle and 386 goats residing on farms situated across Zhejiang Province, China. These farms were distributed across four and five cities for cattle and goats, respectively (Figure 1; Table 3). The farms were selected solely based on the owners' consent to participate and the accessibility of the animals for sampling purposes. The number of collected specimens accounted for 20–30% of total animals in each farm. Each fecal sample was promptly collected from the ground following defecation using sterile disposable latex gloves and placed into individually labeled sterile tubes. These tubes were promptly transported to our laboratory and kept in a cooler packed with ice for no more than 48 h, ensuring their freshness. The samples were subsequently stored at 4°C until they were ready for processing. All animals were in excellent health throughout the sampling period.

**Table 3:** The infection rate and genotypes distribution of *E. bieneusi* in cattle and goats at different locations in Zhejiang Province, China.

**Hosts**	**Location**	**No. positive/No. sample (%)**	**Subtype (*n*)**
Cattle	Lishui	4/56 (7.1)	I (4)
	Ruian	15/71 (21.1)	I (6), J (4), CHG2 (3), CHG3 (2)
	Yongjia	12/71 (16.9)	I (4), J (2), CHG5 (3), CHG19 (2), ZJC-I (1)
	Yueqing	6/67 (9.0)	J (6)
	Subtotal	37/265 (14.0)	I (14), J (12), CHG2 (3), CHG5 (3), CHG3 (2), CHG19 (2), ZJC-I (1)
Goat	Hangzhou	18/92 (19.6)	CHG5 (7), CHG3 (5), CHG2 (2), COS-II (2), ZJG-I (1), ZJG-II (1)
	Lishui	14/88 (15.9)	CHG5 (10), D (4)
	Ruian	16/69 (23.2)	CHG5 (6), CHG3 (8), Henan III (1), ETMK5 (1)
	Yongjia	12/63 (19.0)	CHG5 (4), CHG3 (6), ZJG-III (1), ZJG-IV (1)
	Yueqing	14/74 (18.9)	COS-II (6), CHG2 (6), ZJG-V (1), ZJG-VI (1)
	Subtotal	74/386 (19.2)	CHG5 (27), CHG3 (19), CHG2 (8), COS-II (8), D (4), Henan III (1), ETMK5 (1), ZJG-I to ZJG-VI (one each)
Total		111/651 (17.1)	CHG5 (30), CHG3 (21), I (14), J (12), CHG2 (11), COS-II (8), D (4), Henan III (1), CHG19 (2), ETMK5 (1), ZJC-I (1), ZJG-I to ZJG-VI (one each)

### DNA extraction

All fecal specimens underwent sieving through an 8.0-centimeter diameter sieve, featuring a pore size of 45 micrometers. Following this, the filtrates were concentrated via centrifugation at 1,500 × g for 10 min. Genomic DNA was extracted directly from 200 milligrams of each processed fecal specimen, utilizing the QIAamp DNA Stool Mini Kit (Qiagen, Hilden, Germany). This extraction adhered strictly to the manufacturer's recommended procedures, incorporating an elevated lysis temperature of 95°C to ensure optimal DNA yield. The extracted DNA was then stored at −20°C in a refrigerator, awaiting its subsequent utilization for PCR analysis.

### PCR amplification

To determine the prevalence and genotypes of *E. bieneusi* in animals, nested PCR was performed to amplify a 389 bp fragment encompassing the ITS region using previously established primers and PCR amplification procedures reported by Buckholt et al. (17). TaKaRa Ex Taq^®^ Polymerase was utilized in this experiment (TaKaRa Bio Inc., Tokyo, Japan). Each PCR round incorporated a positive control, which consisted of DNA from the Peru11 genotype isolated from rodents, and a negative control, which was reagent-grade water. This approach ensured the reliability of the results. The secondary PCR products were visualized through electrophoresis in a 1.5% agarose gel stained with GelRed from Biotium Inc. (Hayward, CA).

### Nucleotide sequencing and analysis

The PCR products positive for *E. bieneusi* were subjected to bidirectional sequencing (Sanger sequencing) by Sangon Biotech Co., Ltd., which is located in Shanghai, China. After sequencing, the sequences were meticulously edited and aligned using DNASTAR Lasergene v7.1.0 and Clustal X v2.1 (http://www.clustal.org/). Subsequently, the genotypes of *E. bieneusi* were determined by searching and aligning the sequences with reference sequences retrieved from the National Center for Biotechnology Information (NCBI) (https://www.ncbi.nlm.nih.gov/) using the BLAST algorithm. According to the established nomenclature system for *E. bieneusi*, only a 243 bp segment of the ITS sequence was retained for the identification of novel genotypes.

### Phylogenetic analysis

To establish the genogroup designation and explore the genetic linkage among the ITS genotypes of *E. bieneusi*, the Neighbor-joining (NJ) approach was employed, utilizing the Kimura-2-parameter model within MEGA 7 software. To validate the reliability of these constructed trees, bootstrap analysis was conducted with 1,000 replicates.

### Statistical analyses

Statistical analyses were conducted utilizing SPSS version 22.0, developed by SPSS Inc. in Chicago, Illinois, USA. The chi-square test was employed to assess the prevalence of *E. bieneusi* among cattle and goats, as well as among various farm groups within each host species. Statistical significance was determined when the *P*-values were < 0.05.

### Nucleotide sequence accession numbers

The nucleotide sequences that were obtained in this study have been submitted to the GenBank database, and they are accessible under the accession numbers ranging from PP623828 to PP623847.

## Results

### Infection rates of *E. bieneusi*

Among the 651 fecal samples analyzed, 111 specimens (17.1%) were positive for *E. bieneusi* according to PCR amplification targeting the ITS region of the *SSU rRNA* gene. The prevalence of *E. bieneusi* infection was higher among goats (19.2%, 74/386) compared to cattle (14.0%, 37/265), although the difference was not statistically significant (χ^2^ = 3.01, *P* = 0.08).

Among the cattle, the highest infection rates were found in Ruian (21.1%, 15/71), followed by Yongjia (16.9%, 12/71), Yueqing (9.0%, 6/67), and Lishui (7.1%, 4/56). Significant differences in infection rates were not observed among the four cattle farms studied (χ^2^ = 7.1, *P* = 0.07).

For goats, the highest infection rates were observed in Ruian (23.2%, 16/69) and Hangzhou (19.6%, 18/92), followed by Yongjia (19.0%, 12/63), Yueqing (18.9%, 14/74), and Lishui (15.9%, 14/88). Significant differences in infection rates were also not detected among the five goat farms examined (χ^2^ = 1.3, *P* = 0.86).

### Genotypes of *E. bieneusi*

The *E. bieneusi*-positive samples were categorized into 17 genotypes, encompassing 10 known and seven novel genotypes. The cattle harbored seven genotypes: I, J, CHG2, CHG5, CHG3, CHG19, and ZJC-I. The goats harbored 13 genotypes: CHG5, CHG3, CHG2, COS-II, D, Henan III, ETMK5, and ZJG-I to ZJG-VI (Table 3).

Among the cattle, 70.1% (26/37) of the positive samples were genotypes I and J. CHG2 and CHG3 had the next highest frequency, accounting for 8.1% (3/37) of the positive samples. The remaining genotypes exhibited low frequencies, with a mere handful of occurrences, including 5.4% (2/37) of CHG3 and CHG19 and 2.7% (1/37) of ZJC-I. Notably, the prevalence of genotypes varied among different cattle farms. For instance, Yongjia Farm harbored five genotypes (I, J, CHG2, CHG3, CHG5, CHG19, and ZJC-I), while Rui'an, Yueqing, and Lishui Farms harbored only one subtype each (Table 3).

In goats, the most prevalent genotype was CHG5, accounting for 36.5% (27/74) of the positive samples. This was followed by CHG3 (25.7%, 19/74), CHG2 (10.8%, 8/74) and COS-II (10.8%, 8/74), and D (5.4%, 4/74) in terms of frequency. The remaining genotypes, Henan III, ETMK5, ZJG-I, and ZJG-VI, were observed in a single sample (1.4%, 1/74), respectively. Variations in genotype distributions were observed among the five goat farms. Specifically, CHG5 was detected on four farms excluding Yueqing, CHG3 was identified on three farms (Hangzhou, Rui'an, and Yongjia), CHG2 and COS-II were present in Hangzhou and Yueqing, and D was found only in Hangzhou. Henan III and ETMK5 were found only in Ruian, ZJG-I, and ZJG-II were found only in Hangzhou, ZJG-III, and ZJG-IV were found only in Yongjia, and ZJG-V and ZJG-VI were found only in Yueqing (Table 3).

### Genetic diversity of *E. bieneusi*

**Figure 2:**
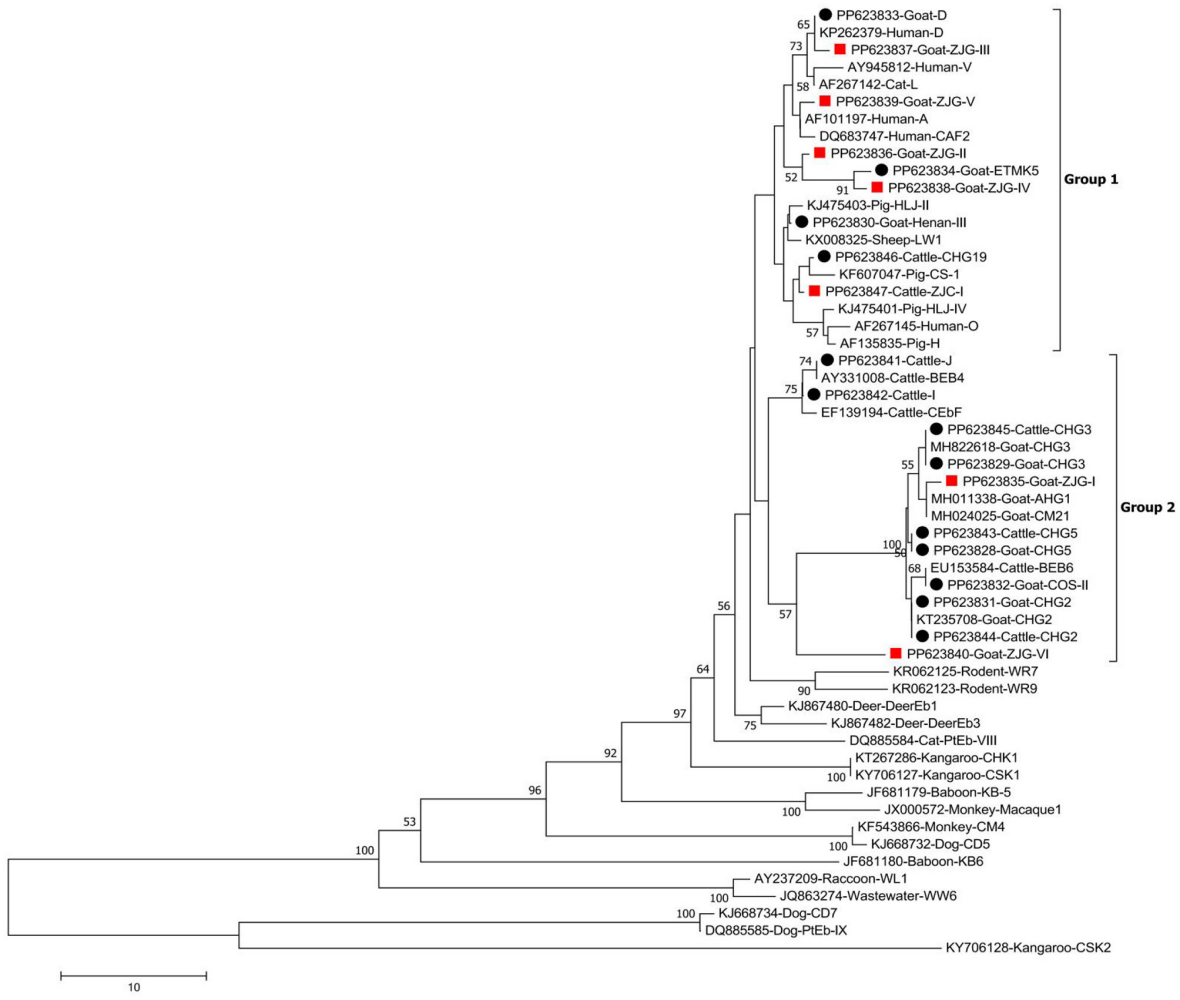
Phylogenetic tree of *Enterocytozoon bieneusi* constructed from ITS sequences. Utilizing the Neighbor-Joining method, the tree was formulated, leveraging the Kimura 2-parameter model as its foundation. To ensure the robustness of the tree, bootstrap values were derived from 1,000 replicates. The tree incorporates black circles and red squares, indicating known and novel genotypes identified in this study, respectively.

Of the 111 identified sequences, seven were novel representatives. Among them, a cattle-derived sequence designated ZJC-I differed by only one base from genotype CHG5. Six goat-derived sequences, designated ZJG-I to ZJG-VI, showed one-base differences from genotypes CM21 (ZJG-I), PigEbITS7 (ZJG-II), and D (ZJG-III) and had 98.8%, 98.8%, and 94.3% similarity with ETMK5 (ZJG-IV), HNM-V (ZJG-V), and LND-I (ZJG-VI), respectively. As shown in Figure 2, the phylogenetic tree analysis categorized all the genotypes into either Group 1 or Group 2 (Figure 2).

## Discussion

Our current study revealed that the overall prevalence of *E. bieneusi* among surveyed animals in Zhejiang Province, China, was 17.1%. Specifically, the prevalence of *E. bieneusi* in cattle was 14.0%, which is considered moderate in comparison to other studies conducted in China, being higher than that in eight provinces, including Anhui (4.2%), Guangdong (12.1%), Hainan (9.9%), Heilongjiang (2.2%), Jiangxi (5.4%), Jiangsu (13.0%), Shandong (2.9%) and Tibet (2.5%), and lower than that in another 12 provinces, including Gansu (22.6%), Hebei and Tianjin (19.4%), Henan and Ningxia (15.1%), Inner Mongolia (25.9%), Jilin (37.6%), Qinghai and Yunan (17.5%), Shanxi (22.4%), Shannxi (19.7%), Shanghai (26.5%), and Xinjiang (28.1%) (Table 1). Combined with previous research findings, the difference in *E. bieneusi* prevalence in cattle among different provinces may result from differences in the grazing conditions. This study focused on the intensive feeding mode, which significantly reduced the direct contact with the external environment and reduced the risk of invasion of external pathogens. On the other hand, the high-density feeding environment may also aggravate the cross-infection among individuals in the farm, thus becoming a potential factor for the difference in infection rate. To deeply explore the true root causes of the differences in infection rates in different regions, more survey data still needs to be extensively collected and thoroughly analyzed. When compared to other countries, the prevalence of *E. bieneusi* in cattle here was higher than most such as South Africa (7.4%), Egypt (6.1%), Thailand (5.0%), America (12.9%), Australia (10.4%), and Turkey (10.3%), but still lower than that in Korea (15.6%), Iran (18.7%) and Brazil (17.4%) (16). Certainly, the variation in infection rates is significant, even within the confines of a single province. For instance, in Heilongjiang Province, the average infection rate was 2.2%, yet the highest recorded rate within the province was 29.0% (18). Additionally, the majority of countries, excluding the United States, have only one or two studies available (16). Therefore, when comparing infection rates across various provinces and countries, exercise caution is crucial.

In our study, the prevalence of *E. bieneusi* among goats was 19.2%, which was comparable to the reported prevalence in Jiangsu (20.0%; 25/125) but lower than those reported in Guizhou (26.7%), Hainan (25.7%), Henan (45.1%), Heilongjiang (21.8%), Ningxia (29.7%), and Shaanxi (32.7%). However, it was higher than the prevalence observed in Anhui (4.6%), Guangdong (17.7%), Inner Mongolia (8.2%), Yunnan (11.8%), and Tibet (9.6%) (Table 2). Only Egypt (13.3%), Thailand (19.2%), Portugal (12.7%), Spain (14.3%), and Peru (2.0%), apart from China, have reported *E. bieneusi* infection in goats (15, 19). However, there is still limited knowledge about *E. bieneusi* infections in goats in numerous countries worldwide. We recommend conducting further studies to elucidate the global prevalence of *E. bieneusi* in goats, as this would serve as a road map for implementing effective public health interventions.

The present study identified 13 genotypes in goats, including seven known genotypes and six novel genotypes. Among the known genotypes, D, Henan III, CHG 5, CHG 3, and CHG 2 have a potential zoonotic transmission capacity. Notably, genotype D, which has garnered significant attention in numerous reports, is present not merely among humans from 40 distinct nations, but also extends to 63 animal hosts across 25 countries (8). In China, this genotype has been found in various animals across multiple regions, including captive red pandas, captive Eurasian wild boars, captive black bears, pet chipmunks, pet red-bellied squirrels, pet birds, sheltered dogs and cats from Sichuan Province (20–24, 67); brown rats, pigs, cats and dogs from Heilongjiang Province (25–27); Asiatic brush-tailed porcupines, bamboo rats, masked palm civets, rodents, cattle and pigs from Hainan Province (12, 28–31); as well as pigs in the study area (32). Therefore, this genotype has significant potential for zoonotic transmission and is capable of cross-species transmission among multiple hosts. Additionally, it has also been detected in some water samples, indicating a broader ecological footprint (5, 8). Meanwhile, there is compelling evidence suggesting the possibility of zoonotic transmission of the genotype Henan-III, which has been detected in humans originating from China and has also been identified in a diverse array of animals, encompassing pigs, whooper swans, bamboo rats, masked palm civets, non-human primates, as well as snakes (29, 33). The present study identified genotype D in four goats and Henan-III in one goat, indicating a need for vigilance regarding these genotypes' potential to infect multiple animal hosts and humans, potentially leading to cross-species transmission.

The CHG2, CHG3, and CHG5 genotypes are the most common genotypes in goats and have been found in almost all goat studies in China (Table 2). More importantly, these genotypes were found in humans, goats and geese in Hainan, China, especially genotype CHG5, which was also detected in cattle and rodents in Hainan, China (12, 13, 31, 34, 35). These findings suggest that the three genotypes have the ability to cause regional zoonotic transmission. Moreover, in addition to goats, these three genotypes were identified in cattle in the present study, which further supports the conclusion that they can cause regional zoonotic transmission. Although humans were not investigated here, these findings could indicate that the three genotypes can spread to each other between cattle and goats in this survey area.

The COS-II genotype was originally identified in sheep from Heilongjiang, China (59). Currently, this genotype is widely reported in sheep and goats from China, but no human or other animal infections have been reported. Therefore, this genotype may be adapted to the specific genotype of sheep and goats. In contrast, the ETMK5 genotype was originally found in *Macaca mulatta* in Vietnam (KJ000018), and also been found in Asiatic brushtailed porcupine in China (29), while we identified this genotype for the first time in goats here. This suggests that it may have a broad host range. However, due to the lack of human case reports, further studies are needed to explore whether this fungus has zoonotic potential.

In addition to the above mentioned human-animal shared genotypes CHG2, CHG3, and CHG5 in cattle, two other genotypes, I and J, have also been identified and exhibit a dominant presence. These genotypes I and J, which were previously thought to be exclusive to cattle, have now been documented in a diverse range of species including bats, birds, canids, felids, primates, pangolins, pigs, rabbits, ursids, rodents, and even humans (8, 16). However, the fact that these two genotypes were identified in only a limited number of humans in a single Chinese study underscores the need for further study to definitively establish their infectivity potential in humans (36).

In the present study, the CHG19 genotype was identified in cattle. This genotype was initially discovered in Chinese goats and has subsequently been identified in goats from multiple regions (Table 2). The presence of CHG19 genotype has also been extensively reported in pigs in China, and it has been identified in masked palm civets from Hainan, grazing horses from Xinjiang, as well as on the surfaces of vegetables and fruits from Henan (28, 30, 37, 38). The identification of the CHG19 genotype in cattle for the first time in this study suggested that it has the potential to infect a wide range of animals. Although CHG19 genotype has not been detected in humans, it should continue to be monitored.

In the present study, seven novel genotypes were discovered. The genotypes ZJG-II to ZJG-V and ZJC-I were grouped under Group 1, whereas the genotypes ZJG-I and ZJG-VI were classified under Group 2. Thus, those genotypes possess the potential for zoonotic transmission, though further epidemiological surveys are required to establish potential animal reservoirs and seek conclusive evidence of zoonotic transmission for these genotypes. The identification of these seven novel genotypes underscores the need for ongoing surveillance and research to gain a deeper understanding of their characteristics and potential risks. This knowledge will play a pivotal role in guiding public health responses and mitigating any potential zoonotic transmission linked to these genotypes.

## Conclusions

This study provides initial evidence of *E. bieneusi* infection in cattle and goats in Zhejiang Province, China. The survey revealed a significant infection rate of 17.1% among the animals, with cattle infected at 14.0% and goats at 19.2%. Furthermore, the study identified a total of 17 genotypes, including 10 known and seven novel genotypes. These findings raise concerns about the potential for zoonotic transmission of these genotypes, which could pose a risk to public health. The discovery of novel genotypes adds valuable genetic diversity information to the existing knowledge base.

## Data Availability

The datasets presented in this study can be found in online repositories. The names of the repository/repositories and accession number(s) can be found in the article/supplementary material.
